# Prenatal diagnosis of fetal respiratory function: evaluation of fetal lung maturity using lung-to-liver signal intensity ratio at magnetic resonance imaging

**DOI:** 10.1002/pd.4469

**Published:** 2014-08-21

**Authors:** Yasuko Oka, Mosfequr Rahman, Chihaya Sasakura, Tomoo Waseda, Yukio Watanabe, Ryota Fujii, Satoru Makinoda

**Affiliations:** Department of Obstetrics and Gynecology, Kanazawa Medical UniversityUchinada, Japan

## Abstract

**Objective:**

The purpose of this retrospective study is to determine the fetal lung-to-liver signal intensity ratio (LLSIR) on T2-weighted images for the prediction of neonatal respiratory outcome.

**Methods:**

One hundred ten fetuses who underwent magnetic resonance imaging (MRI) examination for various indications after 22 weeks of gestation participated in this study. LLSIR was measured as the ratio of signal intensities of the fetal lung and liver on T2-weighted images at MRI. We examined the changes of the ratio with advancing gestation and the relations between LLSIR and the presence of the severe respiratory disorder (SRD) after birth. The best cut-off value of the LLSIR to predict respiratory outcome after birth was calculated using receiver operating characteristic (ROC) curve analysis.

**Results:**

Lung-to-liver signal intensity ratio correlated significantly with advancing gestational age (*R* = 0.35, *p* < 0.001). The non-SRD group had higher LLSIR compared with the SRD group (2.15 ± 0.30 vs. 1.53 ± 0.40, *p* < 0.001). ROC curve analysis showed that fetuses with an LLSIR < 2.00 were more likely to develop SRD [sensitivity: 100%, 95% confidence interval (CI): 52–100%; specificity: 73%, 95% CI 54–88%].

**Conclusion:**

The fetal LLSIR on T2-weighted images is an accurate marker to diagnose the fetal lung maturity. © 2014 The Authors. *Prenatal Diagnosis* published by John Wiley & Sons, Ltd.

## What's already known about this topic?

- In fetuses with normal lung structure, lung-to-liver signal intensity ratio (LLSIR) at MRI increases with advancing gestational age.

## What does this study add?

- LLSIR can be used to predict fetal pulmonary maturity.

## INTRODUCTION

In the process of fetal maturity, the lung is the most important organ that is essential to live in the extra uterine environment. An immature lung requires intensive care immediately after birth. Therefore, it is extremely important to diagnose fetal lung development and maturity accurately before birth. In several previous studies, fetal lung development was determined using conventional^1^–^3^ or three-dimensional^4^,^5^ ultrasonography. Although these techniques are non-invasive means to diagnose pulmonary hypoplasia, they cannot measure the functional maturity of the fetal lung and may be affected by amniotic fluid volume or maternal obesity.

Fetal magnetic resonance imaging (MRI) has been proven to be an efficient and non-invasive tool to examine fetal lung development after the 19^th^ gestational week in vivo.^6^ Several studies used MRI for volume calculation of fetuses with normal lungs,^6^–^8^ and those data can be used to identify fetuses with abnormal lung.^7^,^8^ Although the MRI technique provides detailed anatomic assessments including the estimation of lung volume, it has been used so far mainly to diagnose pulmonary hypoplasia. Several studies evaluated signal intensity of fetal lung to diagnose the lung maturity using T2-weighted MRI.^9^–^12^ The values of signal intensity ratios of lung and liver,^9^–^11^ cerebrospinal fluid^12^ and gastric fluid^11^ were found to increase with advancing gestational age. This phenomenon could be used to predict fetal lung condition.

In previous studies, some authors have mentioned about the relation between lung-to-liver signal intensity ratio (LLSIR) and fetal lung maturity.^9^,^10^ Moshiri M *et al.*^13^ found a linear and significant correlation between LLSIR and gestational age. To our knowledge, no studies have assessed the optimal cut-off value of LLSIR to predict fetal pulmonary maturity prenatally. The purpose of our study is to determine the LLSIR as an accurate prenatal evaluating method of fetal lung maturity and to calculate the cut-off value of LLSIR to predict neonatal respiratory function. In the present study, we retrospectively determined fetal LLSIR on T2-weighted images as a prenatal evaluating method for respiratory outcome.

## METHODS

The subjects of this study were singleton fetuses from 110 pregnant women who underwent MRI examination for various indications after 22 weeks of gestation in the Department of Obstetrics and Gynecology, Kanazawa Medical University Hospital from January 1998 to December 2012. The main indications for MRI of these 110 cases (118 examinations) were as follows: suspicion of fetal congenital diaphragmatic hernia (CDH) (22 cases), placental position abnormality (15 cases), uterine myomas (12 cases), fetal central nervous system abnormality (10 cases), fetal gastrointestinal atresia (8 cases), fetal growth retardation (6 cases), and fetal urogenital abnormality (7 cases) (Table 1). In all cases in which CDH was suspected, the condition was confirmed after birth. All diaphragmatic hernias were confirmed on the left side, and dislocated organs were stomach and small intestine. The calculation of LLSIR in CDH was performed in the non-herniated lung same as in the other cases. Gestational age was confirmed and determined by routine first trimester scans. We received informed consent from parents in accordance with a protocol approved by the local institutional review board. All neonates were born in our hospital. Fetuses with suspected chromosomal anomaly were excluded from this study. To avoid the partial volume effect of MRI and to exclude the extremely small lung, we excluded two cases in which we could not set up over 100 mm^2^ region of interest (ROI). The average size of ROI was 168 mm^2^. A total of 102 fetuses were examined once, and 8 fetuses were examined twice.

**Table 1:** Indications for magnetic resonance imaging (MRI) and neonatal anomalies

Indication for MRI	No. of cases	Neonatal anomaly	No. of cases
Fetal indications			
s/o CDH	22	CDH	22
s/o central nervous system anomaly	10	Hydrocephalus	5
		Other anomalies	5
s/o mass lesion	8	Lymphangioma	2
		Ovarian tumor	1
s/o gastrointestinal atresia	8	Intestinal atresia	5
		Intestinal stenosis	1
s/o urogenital abnormality	7	Renal cyst	2
		Polycystic kidney	1
Fetal growth restriction	6	Ventricular septal defect	1
		Hypospadias	1
s/o abdominal wall defect	3	Abdominal wall defect	3
s/o dilatation of common bile duct	2	(No anomaly)	
Pleural effusion	2	Pleural effusion	2
Indications related to amniotic fluid amount			
Polyhydramnios	1	(No anomaly)	
Oligohydramnios	2		
Placental indications			
Placental positional abnormality	15	(No anomaly)	
Suspicion of placental bleeding	2		
Maternal indications			
Myoma of uterus	12	(No anomaly)	
Other maternal indications	10		

s/o, suspicion of; CDH, congenital diaphragmatic hernia.

Neonates with severe respiratory disorder (SRD) were defined as those who required long-term artificial ventilation for >1 month or who died because of respiratory disorder in spite of the administration of lung surfactant. All neonates without SRD were considered as non-SRD. CDH cases were divided into two groups depending upon the respiratory condition after reparative surgery. For all CDH infants, reparative surgery was performed on the 1^st^ to 3^rd^ day after birth. From these 110 cases, 100 cases were classified as non-SRD, and the other 10 cases were SRD group. Details of these 10 SRD neonates are shown in Table 2.

**Table 2:** Details of the 10 neonates with severe respiratory distress

Indication for MRI	Neonatal condition	Outcome	Gestational week at MRI	Gestational week at delivery	Interval (days) from MRI to delivery
s/o placental bleeding	Premature infant	Death	25	26	1
Oligohydramnios	Premature infant	Death	26	27	7
Oligohydramnios	Premature infant	Death	32	35	21
s/o CDH	CDH	Death	33	35	14
s/o abdominal wall defect	Abdominal wall defect	Alive	35	38	21
s/o CDH	CDH	Death	36	37	7
s/o CDH	CDH	Death	37	39	14
s/o CDH	CDH	Death	40	40	0
s/o CDH	CDH	Death	38	38	1
s/o pleural effusion	Pleural effusion	Death	34	35	10

CDH, congenital diaphragmatic hernia; MRI, magnetic resonance imaging.

**Figure 1:**
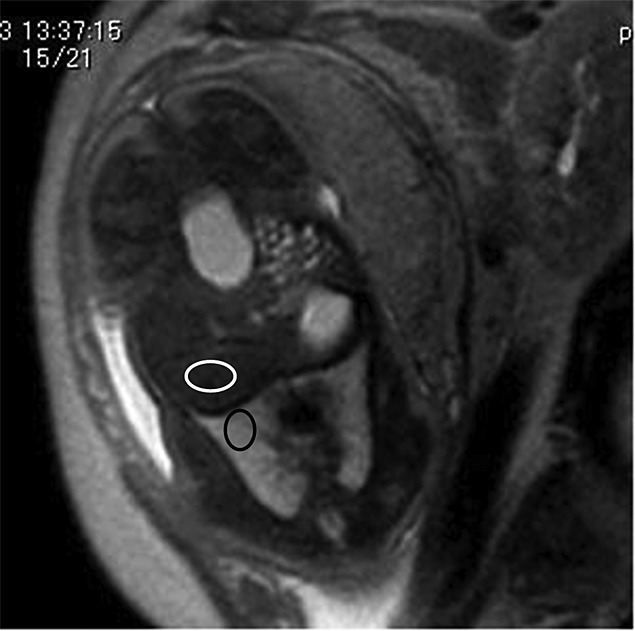
Half-Fourier acquisition single-shot turbo spin-echo magnetic resonance image of fetus at the 37^th^ week of gestation shows regions of interest in both lung (black ellipse) and liver (white ellipse). Chosen areas are homogeneous and free of vascular and tracheal structure

For intra-subject comparison, LLSIR was used. Because of its proximity to the lung and its homogeneity, we chose the liver as a reference organ in this study. The signal intensities of the fetal lung and liver were measured on T2-weighted images, and LLSIR was calculated to measure the fluid content of the fetal lung. MRI was performed in all fetuses with 1.5 T superconductive units (Magnetom Vision, Siemens, Erlangen, Germany and AVANTO, Siemens, Erlangen, Germany) using a phased-array surface coil. After obtaining the horizontal preview of maternal pelvis, half-Fourier acquisition single-shot turbo spin-echo sequences (Magnetom Vision: repetition time (TR) 1000 ms, echo time (TE) 64 ms, field of view (FOV) 350 mm, slice thickness 6 mm, matrix 256 × 112, flip angle 120° and AVANTO: TR 1200 ms, TE 64 ms, FOV 300 mm, slice thickness 5 mm, matrix 256 × 179, flip angle 120°) were oriented in transverse, sagittal, and coronal planes based on the fetal position. We selected the ideal slice in which both the lung and liver were included. In this slice, an ROI was placed within the homogenous portion of the lung and liver (Figure 1). In both lung and liver, three ROIs were placed, and average intensity was calculated to compensate for field inhomogeneity. All calculations of LLSIR were performed by one examiner (Y. O.) to eliminate variations. However, some data were compared with another examiner (M. R.)'s one to confirm the reproducibility. To prevent image distortion due to fetal movement, a sedative agent (Diazepam 5 mg) was administered to the mothers 30 min before the MRI examination. No cases were excluded because of fetal motion.

In the case that the mothers were examined twice, we chose only the earlier examination to investigate the correlation between gestational age and LLSIR. Thirty-eight cases, 30 normal cases, and 8 SRD cases, in which the MRI was performed within 14 days of delivery, were used to examine the relations between LLSIR and the presence of SRD. Moreover, only these cases were used to calculate the suitable cut-off value of the LLSIR for predicting respiratory outcome after birth.The cut-off value of LLSIR for predicting respiratory outcome was assessed by means of receiver operating characteristic (ROC) analysis.

### Statistical analysis

We examined the relations between the LLSIR and gestational weeks for non-SRD cases at first and then the relations between LLSIR and neonatal SRD. The relationship between LLSIR (*y* axis) and gestational age (*x* axis) was evaluated using regression analysis. Analysis of variance and Pearson correlation coefficient were also used to evaluate the data. *P* < 0.05 was considered as the limit for statistical significance. Finally, ROC curve analysis was used to find the suitable cut-off value of LLSIR for predicting respiratory outcome after birth. ROC curve analysis was performed with EZR (Saitama Medical Center, Jichi Medical University, Saitama, Japan), which is a graphical user interface for R (The R Foundation for Statistical Computing, Vienna, Austria).

## RESULTS

**Figure 2:**
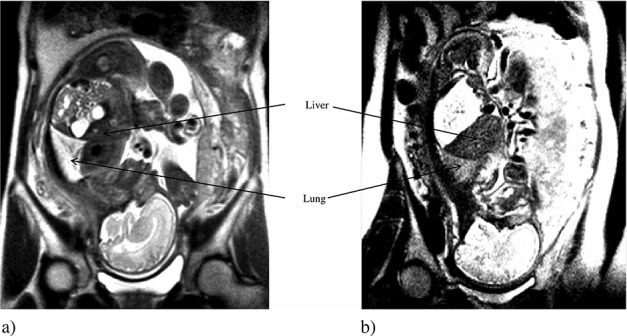
Images of lung and liver on T2-weighted magnetic resonance imaging. (a) Non-severe respiratory disorder (SRD) case at the 32^nd^ week of gestation, lung-to-liver signal intensity ratio (LLSIR) = 2.241. (b) SRD case at the 32^nd^ week of gestation, LLSIR = 1.418

**Figure 3:**
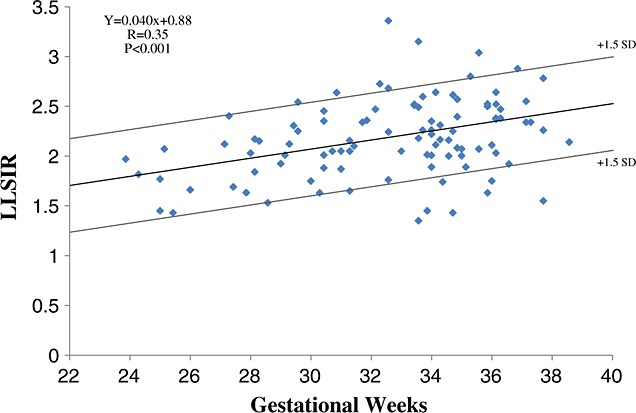
Relation between lung-to-liver signal intensity ratio (LLSIR) and gestational weeks in non-severe respiratory disorder case

**Figure 4:**
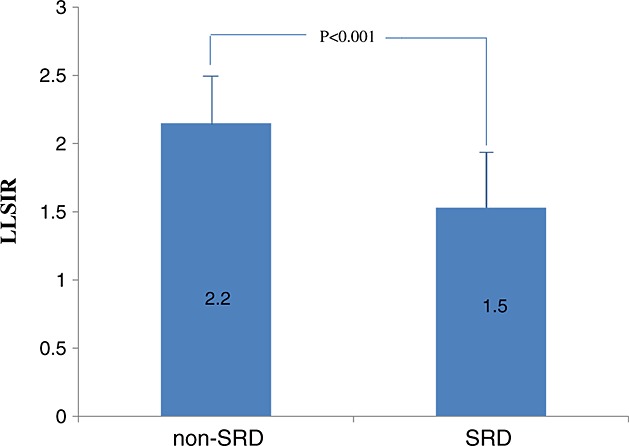
Comparison of mean lung-to-liver signal intensity ratio (LLSIR) between non-severe respiratory disorder (SRD) group and SRD group

Magnetic resonance imaging examinations of the 110 cases (118 examinations) were carried out during the 22^nd^ to 40^th^ week of gestation (32.6 ± 3.6 weeks, mean ± SD). In 18 of 118 examinations, LLSIR was calculated by two examiners to confirm the reproducibility. For examiner 1 (Y. O.), the LLSIR range was 0.860–3.36 (median, 2.04; mean, 1.98). For examiner 2 (M. R.), the LLSIR range was 0.851–3.44 (median, 2.03; mean, 2.00). The concordance was confirmed by Wilcoxon signed-rank test (*p* < 0.001). Figure 2 shows the different images of lung and liver on T2-weighted MRI between non-SRD case and SRD case, respectively. The relationship between LLSIR and gestational age for non-SRD subjects is shown in Figure 3. The best fit for LLSIR of normal lung was represented by the regression line *Y* = 0.040*X* + 0.88 (*r* = 0.35; *p* < 0.001), in which *Y* is LLSIR and *X* is gestational age in weeks (Figure 3). In the eight cases in which MRI was carried out twice, the LLSIR increased with the advancing gestational age (Table 3). The mean gestational age at MRI of non-SRD cases, which intervals from MRI to delivery were within 14 days, was 34.1 ± 3.2 weeks (median 34.8 weeks, range 24–37 weeks). The mean LLSIR was 2.2 ± 0.3 (Figure 4). In non-SRD group, LLSIR varied from 1.5 at the 28^th^ week of gestation to 2.8 at the 37^th^ week of gestation. The mean gestational age at MRI of the SRD cases in which interval from MRI to delivery was within 14 days was 33.7 ± 5.5 weeks (median 35.0 weeks, range 25–40 weeks). In this group, the mean LLSIR was 1.5 ± 0.4, which is significantly lower than the normal group (*p* < 0.001) (Figure 4). The LLSIR of SRD cases varied from 0.9 at the 34^th^ week of gestation to 2.0 at the 36^th^ week of gestation. In fetuses of similar gestational age, the LLSIR of SRD cases was usually less than the LLSIR of non-SRD cases.

**Table 3:** Increment of lung-to-liver signal intensity ratio (LLSIR) in the same cases at various gestational ages

Case number	Gestational week at first MRI	LLSIR	Gestational week at second MRI	LLSIR
1	27	1.63	34	2.01
2	28	2.03	35	2.15
3	27	1.69	34	3.17
4	24	1.97	38	2.15
5	28	1.84	35	1.86
6	26	1.66	36	2.50
7	31	1.65	33	1.85
8	25	1.45	30	1.91

MRI, magnetic resonance imaging.

Nine cases out of 10 SRD cases died after birth. Only one case with LLSIR 1.73 showed favorable outcome in spite of 90 days of hospitalization (Table 2). Although some SRD neonates showed other complications, such as infection, the most important pathology of all SRD neonates was respiratory disorder. Correlation analysis showed that the regression line for LLSIR and gestational weeks in SRD cases was not significant (*r* = 0.15, *p* = 0.726).

**Figure 5:**
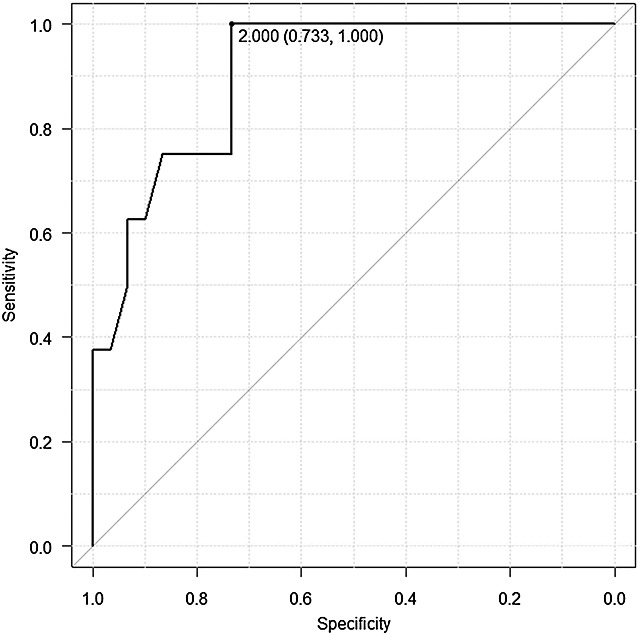
Receiver operating characteristic curve for fetal lung-to-liver signal intensity ratio for predicting postnatal respiratory outcome

The ROC curve on the cases with interval examination-to-delivery <14 days (8 SRD cases and 30 non-SRD controls) obtained by plot at each assumed cut-off value is shown in Figure 5. The area under the ROC curve was 0.904 [95% confidence interval (CI): 0.803–1.01]. It shows that LLSIR is a good indicator for predicting respiratory outcome after birth. On the curve, the best cut-off point is generally chosen at the closest point in which specificity = 1 and sensitivity = 1. Using this cut-off value, we can classify all fetuses in disease predicted group and non-disease predicted group.^14^ A cut-off value of ≤2.0 appeared optimal, providing a sensitivity of 100% (or 8/8) (95% CI = 52–100%) and a specificity of 73% (or 22/30) (95% CI = 54–88%).

## DISCUSSION

When preterm delivery is expected, assessment of fetal lung maturity is important because lung maturity has a great effect on neonatal prognosis. The current effective treatment for respiratory distress syndrome (RDS) is artificial lung surfactant therapy; however, it is ineffective in some cases. Non-invasive assessment of fetal lung maturity has been attempted using ultrasonography.^15^ Gray level histogram width appeared to be a stable and reliable measure of fetal lung maturity using commercial scanners. In that study, the authors found optimal threshold of sonographic parameters related to gray level histogram width to predict neonatal RDS. However, there are different degrees of severity of respiratory distress, and some neonates do not show a response to administration of artificial lung surfactant, suggesting that lung development may be too immature to respond to artificial lung surfactant. Therefore, we evaluated a novel way to predict lung maturity in itself, not related to lung volume or existence of lung surfactant. In this study, we determined the fetal LLSIR of MRI on T2-weighted images as an accurate prenatal evaluative method for fetal lung maturity.

The T2-weighted images of MRI are able to predict the fluid content of fetal organs. The fetal lung exhibit low signal intensity on T2-weighted images because of less fluid content in early pregnancy. A previous study using MRI^10^ found that high intensity points to a substantial amount of fetal lung fluid in the voluminous small airways and alveoli, whereas low intensity indicates the absence of fetal lung fluid. Using this phenomenon, MRI could be considered a useful tool to predict the fetal lung maturity. In contrast to computed tomography, in which tissue density can be expressed as an absolute value in Huns-field units, signal intensity measures in MRI require comparison with a reference structure for standardization.^16^ The ideal conditions of reference structures are sufficient size, close position to the lung, homogeneity and stability through the pregnancy. Although fetal liver signal intensity may change through pregnancy,^17^,^18^ the liver satisfies almost all these conditions. The relation between LLSIR and gestational age has already been studied.^9^–^11^,^13^

**Figure 6:**
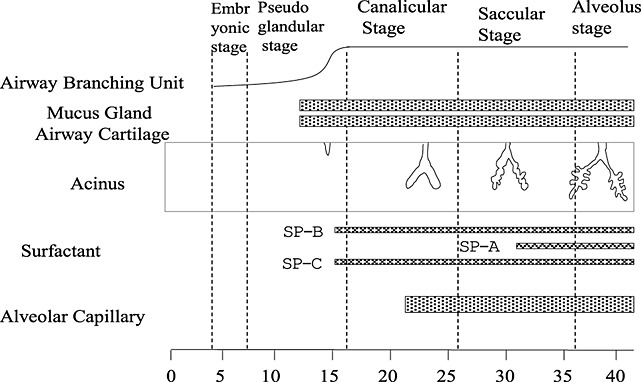
Development process of fetal respiratory system

The development of fetal lungs can be classified into five stages: (a) embryonic stage (4–7 weeks); (b) pseudo-glandular stage (6–17 weeks); (c) canalicular stage (16–28 weeks); (d) saccular stage (26–38 weeks); and (e) alveolus stage (36+ weeks) (Figure 6).^19^ In the process of lung maturation, fetal lung becomes a secretion organ after the pseudo-glandular stage. The future air space becomes filled with Cl-, K^+^, H^+^, rich liquid by the effect of Cl- pump. Lung fluid, which is produced by the epithelial cells, flows via the trachea and is released into the amniotic cavity. The balance of this production and emission is an important factor for the development of the normal lung.^20^ Drainage of lung liquid causes fetal lung hypoplasia on animal experiments.^21^,^22^ Because lung fluid is secreted from the epithelial cells of the fetal lung, the production starts in the late canalicular stage, when the epithelial cells are formed. The quantity of lung fluid increases progressively with fetal growth, and fetal lung becomes organ rich in fluid content. Therefore, high signal intensity indicates a large amount of fetal lung fluid in the airways and alveoli, suggesting lung maturation. In contrast, low intensity suggests a deficiency of lung fluid and suggests pulmonary immaturity. The lung fluid secretion rate increases with the advancement of gestational age because of the development of the pulmonary microvascular and increase of epithelial surface area,^23^ and the immature lungs have fewer and smaller peripheral airspaces and reduced number of bronchi, arteries and veins.^24^ A possible caveat of using MRI to assess fetal lung intensity is that high signal intensity of fetal lung may not actually show the lung liquid in the pulmonary alveoli but rather the interstitial fluid, that is, pulmonary edema. In such a case, LLSIR could be high even if the lung is actually immature.

Higher LLSIR at MRI was observed in normal lung at more advanced gestational ages. This finding is in accordance with the findings of previous reports.^9^,^10^ Our study shows a significant linear correlation between LLSIR and gestational age (Figure 3). This finding is at variance with that of Brewerton *et al.*^9^ which showed a quadratic relationship between LLSIR and gestational age. The reason for the discordance is not clear. However, Moshiri M *et al.*^13^ agreed with our findings: their results demonstrated a significant linear association between LLSIR and gestational age at fetal MRI using.

The results of this study also show significantly higher LLSIR in the non-SRD group than in the SRD group. In other words, good respiratory function is expected by a high LLSIR. The SRD group manifested a low LLSIR throughout pregnancy, yielding a marked difference in signal intensity with the non-SRD group at similar gestational ages.

Finally, we have identified a cut-off value of LLSIR of 2.00 as optimal to predict neonatal occurrence of SRD. However, the 95% CI for the calculated sensitivity and specificity is quite large owing to the small number of cases in our series. Future studies with larger number of subjects are necessary to confirm our finding. Our study had some limitations. Most cases of SRD had been associated with CDH: in such cases, LLSIR may not predict neonatal respiratory function correctly. Two additional cases occurred in extreme premature infants, at gestational ages in which pulmonic maturity is not expected (i.e. <28 weeks). Moreover, we had to exclude cases of lung hypoplasia that do not allow calculation of ROI. However, extremely small lungs cannot carry out the ventilatory function, no matter how mature the lung is. One final limitation of the use of MRI is its cost: our study could be completed because MRI scans are covered by social insurance in Japan; however, cost can be an issue in other countries. Future studies may incorporate LLSIR and fetal lung volume for a more accurate prediction of neonatal respiratory outcome. Moreover, when future studies include more cases at gestational ages above 28 weeks, the information provided by MRI may be clinically more useful.

## CONCLUSION

There is a significant relationship between fetal lung maturation and LLSIR measured by T2-weighed images at MRI, which can be used in the future as a prognostic indicator for neonatal respiratory function.
